# Morphometric Measurement of Cranial Vault Thickness: A Tertiary Hospital Based Study

**DOI:** 10.31729/jnma.3949

**Published:** 2019-02-28

**Authors:** Suraj Thulung, Kajan Ranabhat, Suresh Bishokarma, Dinesh Nath Gongal

**Affiliations:** 1 Department of Neurosurgery, Upendra Devkota Memorial National Institute of Neurological and Allied Sciences, Bansbari, Kathmandu, Nepal; 2 Department of Radiology, Upendra Devkota Memorial National Institute of Neurological and Allied Sciences, Bansbari, Kathmandu, Nepal

**Keywords:** *cranial vault*, *ethnicity*, *Nepalese*, *thickness*

## Abstract

**Introduction:**

The skull's main function is to protect the brain. Total skull bone thickness is the total thickness of diploe and the external and internal tables. The measurement of the human skull based on CT images results are of great practical value in the fields of anatomy, clinical medicine, biomechanics study and head injury analysis. There are few literatures about imaging assisted measurement of the cranial vault thickness while sparse literature among Nepalese population. In this study, we aim to measure the thickness of calvarian bones of and find the difference between gender and ethnic groups.

**Methods:**

This was a descriptive cross-sectional study conducted in our center during a period of 6 months. Patient of age 15 to 50 years with normal CT finding were included in the study. Using the axial view of brain CT, the thickness of cranial vault was measured and recorded in millimeter.

**Results:**

Among 100 patients, 51 were male and 49 were female. Mean thickness of frontal bone, parietal, temporal and occipital bone were 8.02+1.97 mm, 7.04+1.43 mm, 4.71+1.34 mm and 7.98±2.47 mm respectively.

**Conclusions:**

There was no significant difference in cranial vault thickness among sex or ethnical groups in patients of a hospital.

## INTRODUCTION

The skull's main function is to protect the brain. It is comprised of 22 bones, eight of which form the neurocranium and are connected by synarthrodial joints called sutures. Most of these cranial bones are categorized as flat bones and can be identified by their layered bone structure where a cancellous bone layer, called diploe, is sandwiched between two layers of dense cortical bone (cortex).^1^

Most studies measuring skull thickness were done in autopsy.^2–4^ There are few literatures about imaging assisted measurement of the cranial vault thickness while sparse literature among Nepalese population.^5^

In this study, we aim to measure the thickness of calvarial bones and correlate between gender and ethnic groups.

## METHODS

This was a descriptive cross-sectional study conducted in our center during a period of 6 months. Ethical approval was taken from IRC of Upendra Devkota Memorial National Institute of Neurological and Allied Sciences. Patient of age 15 to 50 years with normal CT finding were included in the study. Patient with chronic hydrocephalus, hyperparathyroidism, acromegaly, osteopetrosis, chronic dilantin ingestion, brain tumor, fibrous dysplasia, metastases were excluded.

CT examination of Bone window images of the skull was taken using Siemens 16 Slice, German device; skull bone thickness was assessed by only one consultant radiologist (K.R) for all cases. Using the axial view of brain CT, the thickness of frontal bone was measured in midpoint of coronal suture and nasion, the thickness of the temporal bone is measured in its anterior part and the occipital bone midway between right mastoid bone and internal occipital protuberance while parietal bone was measured in parietal eminence and recorded in millimeter. The results of all these measurements were presented in detail with descriptive statistics, reported as mean, median, range, and standard deviation. Data were analyzed in SPSS version 23. Proportion and mean were deduced for categorical data and continuous variables respectively.

## RESULTS

Among 100 patients, 51 were male and 49 were female (Table 1) Median age was 37.5±0.42 years with minimum age of 15 years and maximum age of 50 years. Median age of male population was 36 years and female population was 38 years. Mean thickness of frontal bone was 8.02±1.97 mm with minimum thickness of 4.7 mm and maximum thickness of 14.7 mm (Table 2). Similarly, mean thickness of parietal bone was 7.04±1.43 mm with minimum thickness of 4.2 mm and maximum thickness of 10.6 mm. Likewise, mean thickness of temporal bone was 4.71±1.34 mm with minimum thickness of 2.3 mm and maximum thickness of 8.8 mm. Moreover, mean thickness of occipital bone was 7.98±2.47 mm with minimum thickness of 4.2 mm and maximum thickness of 20.4 mm. Cranial vault thickness among different ethnicity was studied (Table 3).

**Table 1.** Comparison of cranial vault thickness among gender.

Vault measured	n	Mean	SD
FBT	Male: 51	7.72	1.71
	Female: 49	8.84	2.18
PBT	Male: 51	7.48	1.48
	Female: 49	6.57	1.23
TBT	Male: 51	4.77	1.24
	Female: 49	4.65	1.44
OBT	Male: 51	8.78	2.82
	Female: 49	7.14	1.72

SD: Standard deviation, FBT: Frontal Bone thickness, PBT: Frontal Bone thickness, TBT: Temporal bone thickness, OBT: Occipital bone thickness.

**Table 2.** Cranial vault thickness measurement.

Vault measure	n	Minimum	Maximum	Mean	SD
FBT	100	4.7	14.7	8.02	1.97
PBT	100	4.2	10.6	7.04	1.43
TBT	100	2.3	8.8	4.71	1.34
OBT	100	4.2	20.4	7.98	2.47
SD: Standard deviation, FBT: Frontal Bone thickness, PBT: Frontal Bone thickness, TBT: Temporal bone thickness, OBT: Occipital bone thickness, Confidence interval at 95%.					

**Table 3.** Cranial vault thickness among different ethnicity.

Vault measured	Ethnicity	n	Mean	SD
FBT	Aaryan	57	7.9	2.0
	Mangolian	43	8.6	1.2
PBT	Aaryan	57	7.0	1.4
	Mangolian	43	7.2	1.1
TBT	Aaryan	57	4.7	1.3
	Mangolian	43	4.3	0.8
OBT	Aaryan	57	7.9	2.5
	Mangolian	43	8.2	1.4

## DISCUSSION

Nepal is a beautiful Himalayan country with diverse cast and ethinicity.^6^ There are few literatures about imaging assisted measurement of the cranial vault thickness in Nepalese population.^5,7^ The measurement of the human skull based on CT images results are of great practical value in the fields of anatomy, clinical medicine, biomechanics study and head injury analysis.^8^ Most studies measuring skull thickness were done in autopsy.^2–4^ Thickness of cranial bone is related to the severity of traumatic brain injury. As skull thickness increases, the peak accelerations required to reach a defined peak shear stress threshold in the brain also increases for all impact durations.^9^ Moreover, it may be also helpful in reconstructive plastic surgeries as skull is a frequently used site of bone graft harvest. The data obtained about calvarial thickness study in human population may be useful for researchers, anatomists, anthropologists, surgeons and manufacturers of surgical screws. Pathologically, bone thickness is shown to increase in chronic dilantin ingestion,^10^ hyperparathyroidism,^11^ acromegaly,^12^ meningioma.^13^ Normal thickness of cranial vault among Nepalese population should be known before commenting about the pathological thickness. In our study, conducted among 100 individuals, mean thickness of frontal bone was 8.02±1.97 mm. Similarly, mean thickness of parietal bone was 7.04±1.43 mm. Mean thickness of temporal bone was 4.71±1.34 mm. Moreover, mean thickness of occipital bone was 7.98±2.47 mm. Our finding was similar to the study done by Baral P et al,^7^ among 100 individuals, the mean and standard deviation of thickness of frontal bone was calculated as 5.8±2.1 mm, Parietal bone as 5.4±2.2 mm and Occipital bone as 8.6±2.9 mm.

Li H et al.^14^ analyzed the thickness and the breadth of human skull and found that females tend to have thicker skulls than their male counterpart. Similarly, Ross MD et al.^15^ investigated skull thickness of black and white races and found that white women have the thickest and white men the thinnest skulls. Both the observations were contrasting to our result. Hwang et al.^16^ carried out thickness mapping of the parietal bone in Korean adults and concluded that the parietal bone tended to be thicker towards the Lamda point than at the coronal suture area. Hatipoglu et al. found sexual dimorphism in all craniometric data observed positive correlation between body mass index and diploeic thickness. However, the present study supports the inference of Baral P et al.^7^ finding that Nepalese calvaria showed no sexual dimorphism. Cortical thickness of thin bone is difficult to quantify due to the resolution limitation of clinical CT scans. Thickness measurements of structures thinner than 2.5 mm are overestimated.

This study bears the standard limitation of retrospective study. Consideration of different geography of country with diverse ethnic group was not sufficient in our study.

## CONCLUSIONS

There was no significant difference in cranial vault thickness among sex or ethnical groups in patients of a hospital.
